# Spatial organization of DNA sequences directs the assembly of bacterial chromatin by a nucleoid-associated protein

**DOI:** 10.1074/jbc.M117.780239

**Published:** 2017-03-18

**Authors:** Aleksandre Japaridze, Sylvain Renevey, Patrick Sobetzko, Liubov Stoliar, William Nasser, Giovanni Dietler, Georgi Muskhelishvili

**Affiliations:** From the ‡ Laboratory of Physics of Living Matter, EPFL (École Polytechnique Fédérale de Lausanne), CE 3 316 Lausanne, Switzerland,; § Jacobs University, D-28759 Bremen, Germany,; ¶ UMR5240 CNRS/INSA/UCB, Université de Lyon, F-69003 INSA Lyon, Villeurbanne F-69621, France, and; ‖ Agricultural University of Georgia, 240 David Aghmashenebeli Alley, 0159 Tbilisi, Republik of Georgia

**Keywords:** atomic force microscopy (AFM), bacterial genetics, DNA binding protein, DNA topology, protein-DNA interaction, DNA binding sites, bacterial chromatin, nucleoid-associated protein H-NS, nucleoprotein filaments

## Abstract

Structural differentiation of bacterial chromatin depends on cooperative binding of abundant nucleoid-associated proteins at numerous genomic DNA sites and stabilization of distinct long-range nucleoprotein structures. Histone-like nucleoid-structuring protein (H-NS) is an abundant DNA-bridging, nucleoid-associated protein that binds to an AT-rich conserved DNA sequence motif and regulates both the shape and the genetic expression of the bacterial chromosome. Although there is ample evidence that the mode of H-NS binding depends on environmental conditions, the role of the spatial organization of H-NS-binding sequences in the assembly of long-range nucleoprotein structures remains unknown. In this study, by using high-resolution atomic force microscopy combined with biochemical assays, we explored the formation of H-NS nucleoprotein complexes on circular DNA molecules having different arrangements of identical sequences containing high-affinity H-NS-binding sites. We provide the first experimental evidence that variable sequence arrangements result in various three-dimensional nucleoprotein structures that differ in their shape and the capacity to constrain supercoils and compact the DNA. We believe that the DNA sequence-directed versatile assembly of periodic higher-order structures reveals a general organizational principle that can be exploited for knowledge-based design of long-range nucleoprotein complexes and purposeful manipulation of the bacterial chromatin architecture.

## Introduction

The bacterial nucleoid is a highly organized dynamic entity undergoing structural changes during the growth cycle (1, 2). These structural changes are thought to involve the binding effects of abundant nucleoid-associated proteins (NAPs)^5^ that are produced in a growth-phase-dependent manner and stabilize distinct three-dimensional structures of the DNA (3–5). H-NS (heat-stable nucleoid-structuring protein) is one of the major NAPs (6) present ubiquitously during the bacterial growth cycle and is regarded as a universal gene silencer (7). H-NS not only regulates the expression of genes during the bacterial growth cycle (8) but also is crucially involved in regulation of horizontally acquired genes, the pathogenicity islands, and virulence gene clusters in a wide range of bacterial pathogens (9–16).

The superhelical state of the chromosomal DNA changes during bacterial growth and adaptation to an altered environment (17). H-NS was shown to play an important role in both the regulation of supercoiling response of genomic transcription (8) and the structural organization of the nucleoid (18, 19). H-NS binding sites are distributed in a non-random manner in the genome (3, 20), and in addition, the binding of H-NS varies with DNA topology (21–23), showing preferences for curved DNA (24–27). An AT-rich sequence motif was identified as a characteristic feature of high-affinity H-NS-binding sites (28). On binding to DNA, H-NS can bridge two double helices within an apparently rigid nucleoprotein filament (29, 30). It is assumed that binding of H-NS is nucleated at high-affinity sites and then spreads along the DNA (5, 22, 28). In keeping with this notion, during the bacterial growth cycle the H-NS nucleoprotein complex appears to expand from the initial binding loci in both directions along the genomic DNA, whereby this spreading mechanism has been implicated in gene silencing (20, 31). H-NS can polymerize also on a single DNA duplex leading to its apparent stiffening (32), yet it is the DNA bridging mode facilitated by Mg^2+^ ions that has been primarily implicated in both “caging” and stalling of the transcription machinery by H-NS (33–35). The DNA bridged by H-NS can adopt an interwound configuration consistent with a right-handed plectoneme (5, 28, 30). Furthermore, H-NS was shown to efficiently constrain negative supercoils in the DNA *in vitro* (36) and modulate the DNA topology *in vivo* (8).

Whereas there is ample evidence that changing environmental conditions can determine the binding and polymerization mode of H-NS on the DNA, the role of the spatial pattern of high-affinity H-NS binding sequences in the ensuing three-dimensional structure remains unknown. To answer this question we used circular DNA constructs carrying identical H-NS binding sequences in different spatial arrangements. We demonstrate that distinct arrangements of binding sequences not only result in distinct morphology and periodicity of the H-NS nucleoprotein structure but also a different capacity to constrain supercoils and compact the DNA. We thus provide the first experimental evidence that variation in linear arrangement of identical binding sequences for an abundant NAP leads to organization of different long-range DNA structures.

## Results

**Figure 1.**
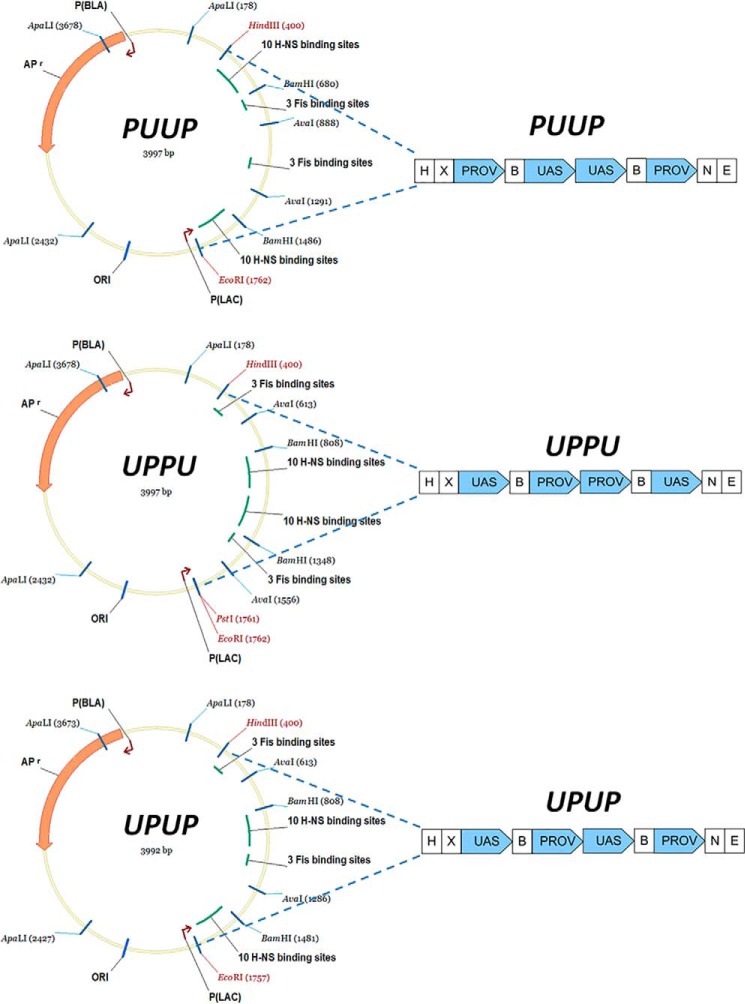
**DNA maps of the three circular DNA constructs used in the experiments.** *Linear dotted lines* indicate the region of the inserted construct. *U* stands for genomic DNA from the *tyrT* UAS sequence, and *P* stands for *proV* NRE sequence respectively. The full size of the constructs is 4 kb, and the inserts are 1.3 kb. *H*, *X*, *N*, *B*, and *E* indicate the cleavage sites for HindIII, XbaI, NheI, BamHI, and EcoRI restriction endonucleases, respectively.

To investigate the impact of spatial organization of H-NS binding sites on higher-order nucleoprotein complex formation, we generated three circular constructs containing the regulatory DNA sequences from the *Escherichia coli proV* and *tyrT* gene promoters in different spatial arrangements. *ProV* is the first structural gene of an operon encoding a transport system involved in sensing of elevated osmolarity. The negative regulatory element (NRE) of this operon partially overlaps the 5′-end of *proV* gene and contains multiple H-NS binding sites including two identical high-affinity sites (*K_d_* values between <15 nm and 25 nm) separated by ∼10 helical turns of DNA. H-NS binds cooperatively at this region, silencing the operon. Whereas the sequence periodicity of the *proV* NRE favors plectonemic coiling of DNA, nucleation of H-NS binding at the helically phased high affinity sites facilitates the bridging of duplexes (22, 35). In contrast, the upstream activating sequence (UAS) of the *tyrT* (tyrosyl tRNA) gene promoter is characterized by bending anisotropy favoring toroidal coiling (37), which potentially would impede the bridging of DNA duplexes by H-NS. So far, no high-affinity binding sites for H-NS have been reported in *tyrT* UAS. We thus constructed plasmids containing the *proV* NRE sequences (denoted as “P”) and the *tyrT* UAS sequences (denoted as “U”) in three different tandem arrangements, PUUP, UPUP, and UPPU, in which the proximal high-affinity H-NS binding sites of two P elements were separated by 941, 544, and 147 bps of DNA, respectively (Fig. 1). These three plasmid constructs were used to visualize the nucleoprotein structures generated after incubation with increasing concentrations of H-NS by atomic force microscopy (AFM). To avoid complications of visualizing distinct structures arising from tangling of the supercoiled DNA, the plasmid constructs were uniquely nicked with Nt.BspQI nuclease (see “Experimental procedures”). All experiments (except AFM on a APTES-modified surface) were carried out in the presence of Mg^2+^ ions facilitating the DNA bridging by H-NS (34).

### Global organization of the DNA by H-NS

**Figure 2.**
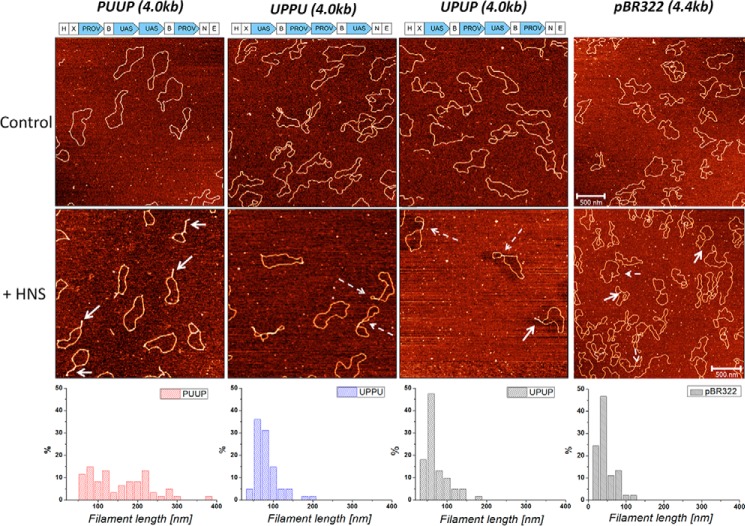
**AFM images of control and H-NS bound circular DNA constructs (PUUP, UPPU, and UPUP) and pBR322 DNA.** The *lower panels* show the respective distributions of maximal bridged filament length. *White continuous arrows* indicate bridge-loop structures; *dashed arrows* indicate loop-bridge-loop structures. The H-NS:DNA molar ratio was 13:1. PUUP construct formed the longest filaments exclusively on bridge-loop structures; UPPU formed shorter filaments with exclusively loop-bridge-loop structures; pBR322 DNA, which did not contain any strong binding sites for H-NS formed shortest filaments. 500 nm scale bar. *H*, *X*, *N*, *B*, and *E*indicate the cleavage sites for HindIII, XbaI, NheI, BamHI, and EcoRI restriction endonucleases, respectively.

**Figure 3.**
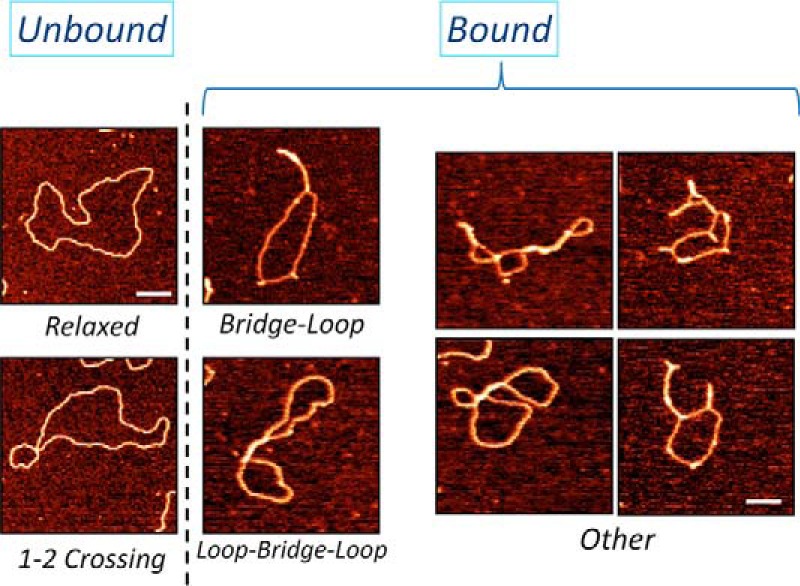
**Close-up AFM images of various structures formed by H-NS DNA complexes.** Control DNA formed either completely relaxed structures or structures with one or two self-crossings (*Unbound*); H-NS bound DNA formed predominantly either bridge-loop or loop-bridge-loop structures (*Bound*). Other more complex structures were also present in H-NS-bound samples, although their frequency was low compared with BL or LBL structures. *Scale bar*, 100-nm.

In general, three classes of structures indicating an extensive bridging of DNA by H-NS were observed with all constructs. Two of the observed classes of bridged complexes displayed clearly distinct architectures, denoted as bridge-loop (BL) and loop-bridge-loop (LBL) structures (indicated by *arrows* in Fig. 2), whereas the third class comprised more complex structures (representative examples of the observed structures are shown in Fig. 3). Notably, statistical measurements showed that these three classes of structure were formed in different proportions depending on both H-NS concentration and on the construct used (Table 1). With the PUUP construct harboring two *proV* NRE regions separated by two copies of the *tyrT* UAS sequences, the LBL structures prevailed over BL structures at low and intermediate H-NS concentrations, whereas at the highest used H-NS concentration (H-NS:DNA molar ratio of 13:1) the BL structures (∼50%) were predominantly observed (Table 1). Also with the UPUP construct, the LBL structures prevailed at low and intermediate H-NS concentrations, yet in contrast to PUUP at the highest used H-NS concentrations, the BL and LBL structures formed with equal efficiency. Interestingly, the third construct, UPPU, in which the two adjacent *proV* NRE regions were flanked by *tyrT* UAS, formed exclusively LBL structures, attaining their maximum (∼17%) at the highest used H-NS concentration (Table 1). For these three constructs the overall amount of H-NS-bridged complexes was also different, being lowest for UPPU (56%, comparable with pBR322 control), slightly higher for UPUP (∼65%), and highest for PUUP (∼80%). These data suggested that whereas H-NS can bridge the DNA duplexes in all three constructs, the spatial arrangement of P sequences with high-affinity binding sites determines both the efficiency of binding and the preferred morphology of ensuing DNA structures.

**Table 1:** **Structural changes of nucleoprotein complexes formed on various constructs with increasing concentrations of H-NS** The standard error bars are calculated by the variation in the number of individual DNA structures observed on different AFM images for a given H-NS-DNA ratio.

*H-NS:DNA*	Relaxed DNA	1-2 crossing	Bridge-loop	Loop-bridge-loop	Other	Total number	Unbound	Bound
**PUUP (4 kb)**								
Control	58 ± 5%	15 ± 5%	4 ± 2%	3 ± 2%	20 ± 5%	144	73 ± 5%	27 ± 5%
6:1	46 ± 5%	24 ± 5%	3 ± 2%	8 ± 5%	18 ± 5%	157	71 ± 5%	29 ± 5%
9:1	42 ± 5%	9 ± 4%	7 ± 3%	20 ± 5%	23 ± 5%	158	51 ± 5%	49 ± 5%
13:1	21 ± 5%	1 ± 2%	49 ± 5%	6 ± 5%	22 ± 5%	156	22 ± 5%	78 ± 5%

**UPUP (4 kb)**								
Control	58 ± 5%	21 ± 5%	3 ± 2%	3 ± 2%	15 ± 5%	134	79 ± 5%	21 ± 5%
6:1	42 ± 5%	7 ± 3%	8 ± 3%	17 ± 5%	26 ± 5%	133	49 ± 5%	51 ± 5%
9:1	40 ± 5%	4 ± 2%	9 ± 3%	17 ± 5%	30 ± 5%	116	44 ± 5%	56 ± 5%
13:1	25 ± 5%	11 ± 4%	23 ± 5%	22 ± 5%	19 ± 5%	96	36 ± 5%	64 ± 5%

**UPPU (4 kb)**								
Control	48 ± 5%	25 ± 5%	0 ± 2%	6 ± 5%	21 ± 5%	142	73 ± 5%	27 ± 5%
6:1	54 ± 5%	11 ± 5%	2 ± 2%	11 ± 5%	22 ± 5%	170	65 ± 5%	35 ± 5%
9:1	47 ± 5%	13 ± 5%	3 ± 2%	15 ± 5%	22 ± 5%	152	60 ± 5%	40 ± 5%
13:1	31 ± 5%	13 ± 5%	1 ± 2%	17 ± 5%	38 ± 5%	151	44 ± 5%	56 ± 5%

**pBR322 (4.4 kb)**								
13:1	20 ± 5%	22 ± 5%	10 ± 5%	14 ± 5%	34 ± 5%	135	42 ± 5%	58 ± 5%

### Properties of the H-NS-bridged filaments

To get insight into the peculiar organization of the bridged filaments formed on constructs with different arrangements of H-NS binding sequences, we measured various shape parameters. One of the shape parameters we measured was the persistence length *l_p_*, a basic mechanical property of DNA quantifying its flexibility. As expected, the persistence lengths of the unbound PUUP, UPUP, and UPPU DNA constructs provided fairly similar values of *l_p_* ∼ 60 ± 2 nm. The addition of H-NS significantly affected the persistence lengths for PUUP and UPPU constructs (Table 2). Furthermore, at the highest H-NS concentration used, we obtained significantly lower persistence-length values for UPPU (*l_p_* = 44 ± 2 nm) compared with both UPUP and PUUP (*l_p_* = 54 ± 2 nm and *l_p_* = 64 ± 2 nm, respectively).

**Table 2:**
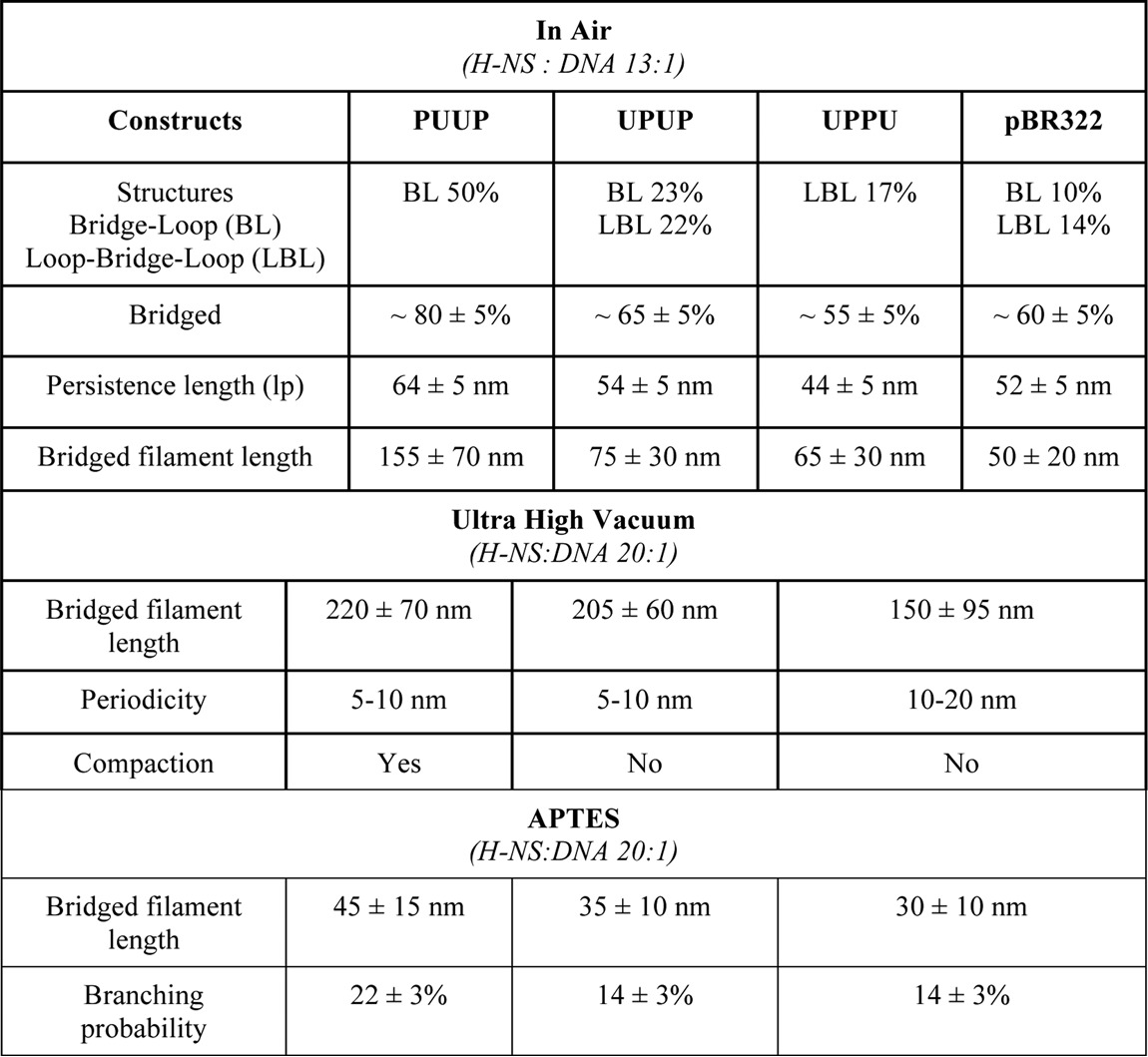
**Comparison of various shape parameters of DNA constructs at high H-NS concentration(H-NS:DNA ratio - 13:1 and 20:1) and different deposition methods (MgCl_2_ and APTESmodified mica surface)** For AFM measurements in air up to 170, in ultra high vacuum up to 45and on APTES up to 100 individual molecules were analysed.

**Figure 4.**
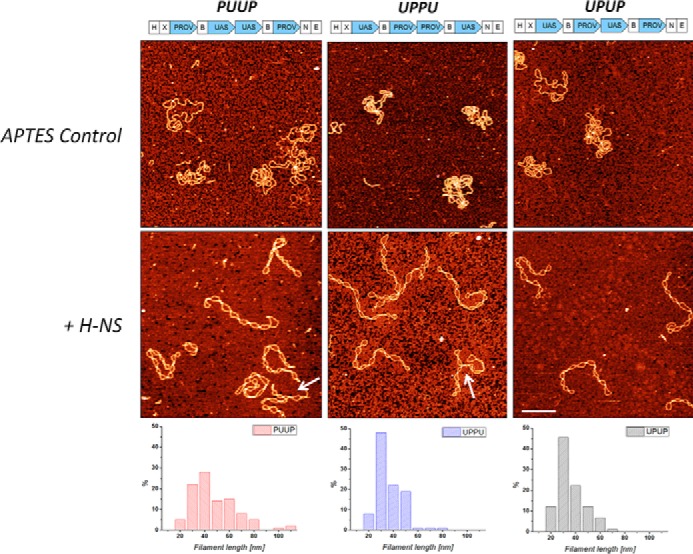
**AFM images of control and H-NS bound circular DNA constructs (PUUP, UPPU, and UPUP) deposited on APTES-treated mica for a H-NS:DNA molar ratio of 20:1.** The *lower panels* show the respective distributions of maximal bridged filament length. Control DNA deposited on APTES surface is compact with many self-crossings. The addition of H-NS results in several bridged regions between DNA strands, stabilizing DNA plectonemes. In certain cases DNA is branched, with the branching probability highest for PUUP constructs (indicated with *white arrows*). Similarly, as is the case in Fig. 2, PUUP constructs formed the longest, whereas UPPU formed the shortest bridged filaments. *Scale bar*, 500 nm. *H*, *X*, *N*, *B*, and *E* indicate the cleavage sites for HindIII, XbaI, NheI, BamHI, and EcoRI restriction endonucleases, respectively.

**Figure 5.**
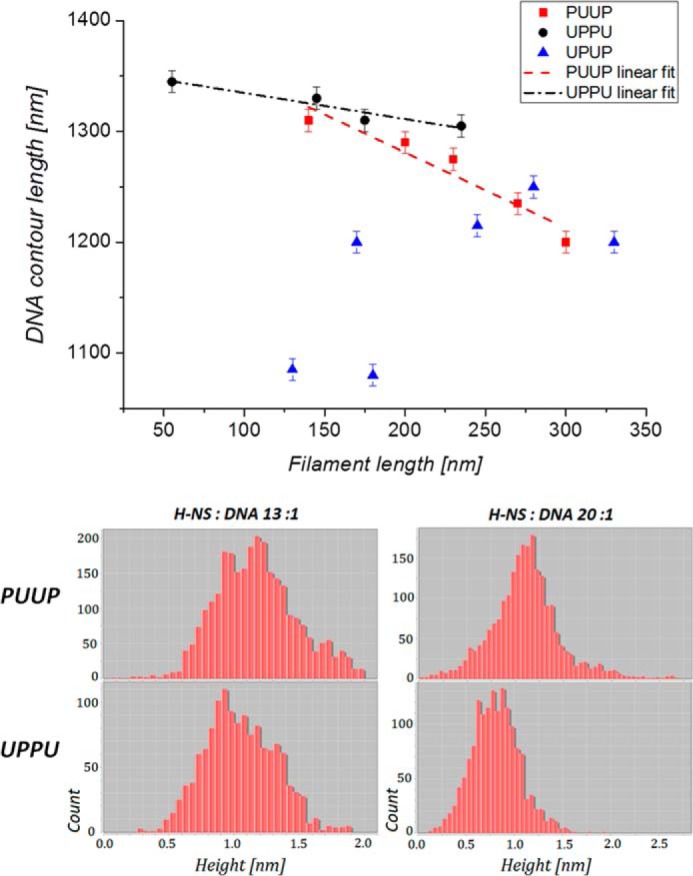
**Shape parameters of the bridged filaments.** *A*, DNA contour length dependence on H-NS-bridged filament length. PUUP construct shows a clear inversely linear dependence on filament length, indicating a linear shortening of the contour length as the function of H-NS-bridged filament growth (on average a 30-nm DNA contour length compaction for every 100 nm of DNA filament). This dependence is less pronounced for UPPU (on average a 15-nm DNA contour length compaction for every 100 nm of DNA filament) and absent in the case of UPUP. *B*, measured height distributions along H-NS-bridged filament for PUUP and UPPU constructs. Note that for both 13:1 and 20:1 H-NS-to-DNA molar ratios, the average height along a bridged filament is always larger for PUUP than for a UPPU construct.

Measurements of the bridged filament lengths (*L_bf_*) again demonstrated differences between the three constructs (Table 2). The bridged filament formed on UPUP showed a slightly higher average length (*L_bf_* = 75 ± 30 nm) compared with UPPU (*L_bf_* = 65 ± 30 nm), whereas those formed on PUUP had the largest average length (*L_bf_* = 155 ± 70 nm; see Fig. 2, *lower panel*). However, all these exceeded the lengths measured for the pBR322 control. We verified these differences by imaging the nucleoprotein complexes on the APTES-modified mica surface, which was due to strong charge interactions kinetically trapping the complexes and, thus, better preserving their three-dimensional configuration (38–41). When deposited on the APTES-treated surface, the morphology of the nicked DNA changed significantly. Despite this, the observed differences between the H-NS-DNA complexes formed on various constructs were similar (Fig. 4; Table 2), indicating that these differences were robust and independent of the deposition method used. We also measured the propensity of branching for APTES-deposited DNA by defining the branching probability as the ratio between the number of DNA molecules having three ends (branches) to the total number of DNA molecules (branching = *N*_3ends_/*N*_total_; typical branched molecules are indicated by *white arrows* in Fig. 4). We found that the PUUP construct showed the highest propensity of branching (Table 2). Thus, the PUUP and UPPU constructs with opposite arrangements of the P and U sequences bind H-NS with different propensity, favor, respectively, BL and LBL structures, and significantly differ in their average bridged filament lengths (see Table 2). Furthermore, plotting of the bridged filament lengths at the maximal used H-NS concentration (H-NS:DNA molar ratio 20:1) against the plasmid contour length revealed a clear inversely linear dependence for PUUP, which was less pronounced for UPPU but completely absent in the case of UPUP (Fig. 5*A*). Finally, we found that these bridged filaments also demonstrated clear differences in measured height (Fig. 5*B*). Taken together, these measurements with various constructs indicated a difference in DNA packing by H-NS.

### Periodic structure of H-NS bridges

**Figure 6.**
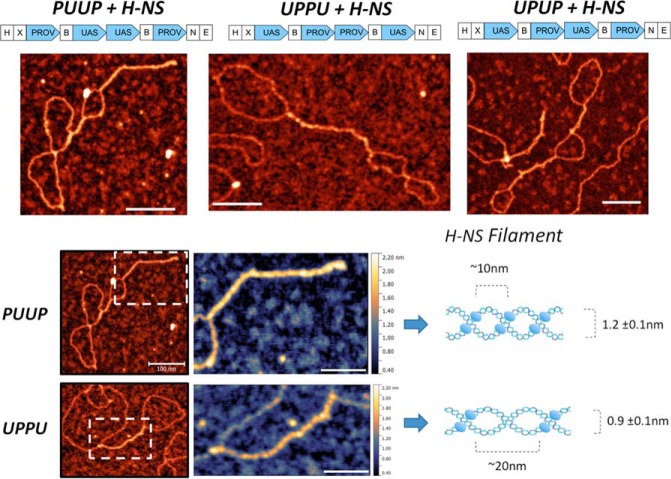
**Ultra high vacuum AFM.** *A*, images of different constructs bound by H-NS for H-NS:DNA molar ratio 20:1. *Scale bars*, 100 nm. *B*, the close-up view of the PUUP- and UPPU-bridged filaments and the corresponding model with measured shape parameters. The bridging H-NS dimers are indicated by *blue ellipsoids*. In this model PUUP binds H-NS with high frequency, resulting in higher periodicity of the bridged filament. In contrast, cooperative binding of H-NS to consecutive high-affinity sites of tandem P elements in UPPU stabilizes a plectoneme with high pitch and lower height and periodicity compared with PUUP DNA. We note that the height difference between the PUUP- and UPPU-bridged filaments might be explained by different protein interaction sites involved in H-NS dimer formation (45). *Scale bars*, 50 nm. *H*, *X*, *N*, *B*, and *E* indicate the cleavage sites for HindIII, XbaI, NheI, BamHI, and EcoRI restriction endonucleases, respectively.

To gain more insight into the packing differences of various H-NS-bridged filaments, we acquired high-resolution data by using ultra high vacuum AFM (Fig. 6*A*). In all constructs the bridged filaments demonstrated a clear periodicity, but the average periodicity was different, ∼5–10 nm for PUUP and UPUP and ∼10–20 nm for UPPU (Table 2; Fig. 6*B*). The measured periodicity values are in reasonable agreement with two different average periodicity values (12.1 ± 2.4 nm and 24.2 ± 4.3 nm obtained previously for H-NS-bridged filaments formed on the linear DNA of phage λ (5).

### H-NS binding distinctly constrains the DNA on different constructs

**Figure 7.**
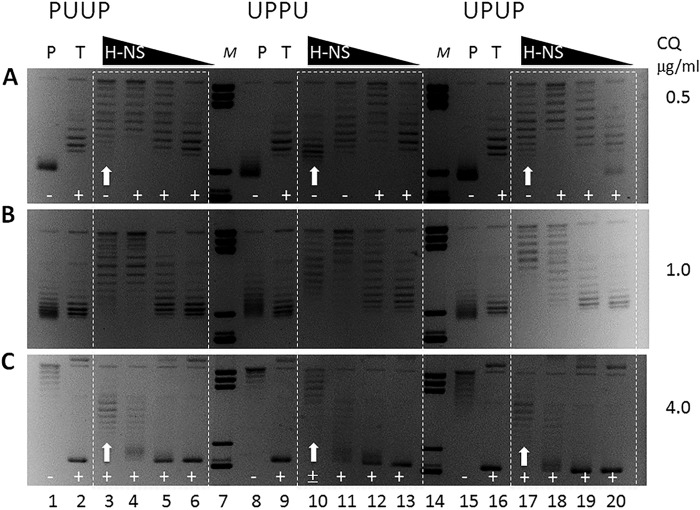
**High-resolution agarose gel electrophoresis of PUUP, UPPU, and UPUP constructs.** The plasmids were incubated in the presence of different H-NS concentrations and relaxed by *E. coli* topoisomerase I. *P*, untreated plasmid; *T*, topoisomerase I treatment; *M*, DNA size marker. *A*, gel electrophoresis at 0.5 μg/ml chloroquine (*CQ*) concentration. Note that under these conditions the plasmids relaxed by topoisomerase I treatment (*T*) migrate as moderately positively (+) supercoiled species in the gel (*lanes 2*, *9*, and *16*). The addition of H-NS has negligible effects at lowest concentrations (*lanes 6*, *13*, and *20*) but with increasing H-NS concentration (*lanes 5*, *12*, and *19*) the migration of plasmids is retarded because they are less positively supercoiled (under the influence of chloroquine) due to protection of negative supercoils by H-NS binding. This effect becomes more conspicuous at higher H-NS concentration for PUUP and UPUP (*lanes 4* and *18*) but not for UPPU (*lane 11*). This is because at this concentration the constraint of UPPU DNA by H-NS is efficient enough to preserve negative superhelicity (despite the influence of chloroquine), and so the population in *lane 11* migrates as negatively (−) supercoiled species. This difference in the efficiency of constraint between UPPU and the other constructs is clearly evident at the highest H-NS concentration (*lanes 3*, *10*, and *17*, indicated by *white arrows*). *B* and *C*, gel electrophoresis at 1 μg/ml and 4 μg/ml chloroquine concentration, respectively.

We asked whether the differences between the constructs in DNA packing measured by AFM could be observed also in solution. Because H-NS binding was shown to constrain negative DNA superhelicity *in vitro* (36), we monitored the distributions of topoisomer populations of supercoiled PUUP, UPPU, and UPUP constructs incubated with increasing H-NS concentrations (see “Experimental procedures”). The preformed complexes were treated with *E. coli* topoisomerase I, which exclusively removes free (unconstrained) negative supercoils, enabling the measurement of “topoisomerase-resistant” superhelicity constrained on binding of H-NS. After deproteinization, the plasmid topoisomer distributions were analyzed by high-resolution agarose gel electrophoresis in the presence of different intercalator (chloroquine) concentrations to reveal the differences in average levels of global negative supercoiling. We observed higher average levels of negative superhelicity for UPPU, indicating that binding of H-NS constrained the DNA supercoils much more efficiently than with PUUP or UPUP (Fig. 7; the effect is most clearly seen at the highest H-NS concentrations indicated by *white arrows*). Indeed, even at the highest chloroquine concentration used, the UPPU DNA remained partially negatively supercoiled, whereas both the PUUP and UPUP DNAs migrated as positively supercoiled species. Thus, UPPU differed from the two other constructs not only in periodicity but also in the extent of supercoiling constrained by H-NS binding.

### H-NS-bridged filaments show differences in protection from restriction endonuclease and DNase I cleavage

**Figure 8.**
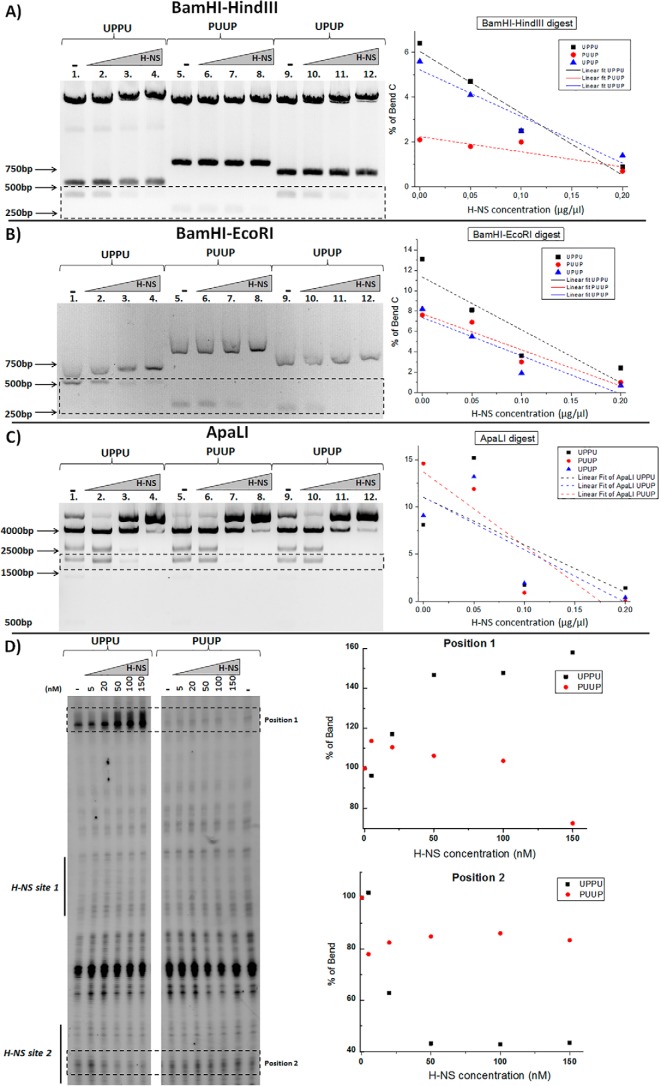
**Probing of H-NS-DNA complexes with restriction endonucleases and DNase I footprinting.** In *A*, *B*, and *C*, the *left panels* show agarose gels with digests; the *right panels* show the change of the relative intensity of specific restriction bands (indicated with *black dotted rectangles on the left panel*) in each digestion reaction. Shown is probing with BamHI-HindIII (*A*), BamHI-EcoRI (*B*), and ApaLI (*C*). When the restriction sites are inside the inserted fragment (HindIII or EcoRI sites inside the UPPU, PUUP, or UPUP DNA) the highest protection rate is observed for the UPPU construct (steepest decay of band intensity). When the restriction sites are in the plasmid backbone (ApaLI restriction sites), the highest protection is observed for the PUUP construct and the weakest for UPPU DNA. *D*, DNase I footprinting of supercoiled UPPU and PUUP constructs at different H-NS concentrations (0–150 nm). H-NS binding sites 1 and 2 are indicated on the sequencing gel image (*left panel*) by *black vertical lines*. H-NS induced hypersensitivity (*Position 1*) and protection in H-NS binding site 2 (*Position 2*) in UPPU (marked by *dotted rectangles*). The *graphs on the right* show the relative intensity of the Position 1 and Position 2 signals at various H-NS concentrations.

To test whether the observed differences were associated with occupation of different DNA regions in various plasmid constructs, we probed the H-NS nucleoprotein complexes with restriction endonucleases cutting the DNA either at the junctions of the U and P elements (BamHI, HindIII, EcoRI) or in the plasmid backbone (ApaLI; see Fig. 1). We found that the UPPU construct demonstrated the highest protection rate of the junction sites, whereas PUUP demonstrated the highest protection with ApaLI endonuclease cutting in the plasmid backbone. With UPPU the ApaLI protection was weakest (Fig. 8, *A–C*). This indicates that in the UPPU construct H-NS polymerizes more efficiently across the inserted UPPU sequence than elsewhere in the plasmid backbone.

To obtain more precise information on the peculiarities of H-NS binding to the P elements in UPPU and PUUP constructs, we carried out DNase I footprinting using a primer hybridizing 72 bp downstream of H-NS binding site 2 in the P element. We found that increasing H-NS concentrations induced a strong DNase I hypersensitivity signature upstream of the two high-affinity H-NS binding sites in UPPU but not PUUP construct. This signature indicated DNA deformation in the region between the two P elements of UPPU (Fig. 8*D*, see *Position 1*). However, the protection of the H-NS binding site 2 was also more pronounced in UPPU than in PUUP (Fig. 8*D*, see *Position 2*).

Taken together our data indicate that not only the overall DNA shape and the level of constrained superhelicity but also the packing, stiffness, extension, height, and directional polymerization of the H-NS-bridged filaments depends on the spatial arrangement of the U and P sequences in the substrate DNA.

## Discussion

H-NS is a major DNA-bridging NAP acting predominantly as a repressor involved in regulation of various cellular functions including bacterial motility, stress response, and virulence (7, 42). The H-NS effects are thought be associated with nucleation of binding and polymerization of the protein on the DNA. In this study we investigated whether DNA sequences harboring different spatial arrangements of natural sequences binding this abundant DNA architectural protein can determine the three-dimensional structure of the ensuing nucleoprotein complex. In our experiments we used various combinations of identical sequences harboring high-affinity H-NS binding sites from the *proV* NRE sequence linked with UAS regions of the *tyrT* promoter characterized by bending anisotropy. The sequences were arranged in three different configurations such that in one construct, two *proV* NRE regions were “sandwiched” between two flanking UAS regions (UPPU). In the second construct these regions were arranged in opposite way (PUUP), whereas in the control construct UPUP individual *tyrT* UAS and *proV* NRE regions were arranged in an alternating manner.

Overall, we could discern three major classes of structures indicating an extensive bridging of DNA by H-NS. In principle, this bridging could be a result of either H-NS coating a single double helix and subsequently capturing the naked “partner” duplex or by H-NS molecules forming initial bridges on binding high-affinity nucleation sites followed by spreading. The third scenario, in which bridging of certain regions occurs in plasmids fully coated by H-NS, seems implausible for two reasons. First, we did not detect any significant differences between the measured heights of the DNA in the unbridged regions of H-NS complexes and the naked control DNA (data not shown). Second, we observed bridging at various H-NS concentrations whereby even at the highest H-NS-to-DNA molar ratio (13:1), when ∼ 60–80% of plasmids demonstrated bridging (Table 1), the used concentration of H-NS was still an order of magnitude below the levels required to cover the entire plasmid DNA.

We observed conspicuous differences between the H-NS nucleoprotein complexes formed on these constructs. These differences were observed by AFM with nicked DNA samples deposited on the surface by various methods both in air and under ultra high vacuum as well as with supercoiled DNA molecules in solution. Thus, notwithstanding the deviation of our experimental conditions from the *in vivo* environment, the observed differences are robust and should be attributed only to peculiar organization of the U and P sequences in the used constructs.

First, we found that binding of H-NS stabilized bridged nucleoprotein complexes of different gross configurations on different constructs. At high H-NS concentrations BL structures were preferentially formed on PUUP and exclusively LBL structures on UPPU, whereas the “intermediate” construct UPUP formed both structures with equal efficiency (Table 2). Secondly, we found that the binding of H-NS and, correspondingly, the average length of the bridged filaments varied significantly between the constructs, being the largest for PUUP. Latter nucleoprotein complexes also demonstrated the highest persistence length and a clear inversely linear dependence between the contour and bridge lengths. Thirdly, whereas H-NS-UPPU-bridged filaments showed a low average height and weak inverse dependence between the contour and bridge lengths, they constrained supercoils more efficiently than complexes formed on PUUP or UPUP. Finally, we observed that the bridged filament periodicity for UPPU construct was larger than for both PUUP and UPUP. Importantly, whereas the constructs with “opposite” arrangements of binding sequences (PUUP and UPPU) differed across all the measured parameters, the control construct UPUP showed intermediate values as expected or was closer to either PUUP or UPPU. For example, the UPUP structures formed by H-NS binding were more akin to UPPU in terms of bridge length distributions and propensity of branching and to PUUP in terms of periodicity and constraint of DNA superhelicity, whereas both the overall H-NS binding (percentage of bridged complexes) and persistence length showed intermediate values (Table 2). Taken together our data indicate that variations of the spatial arrangement of identical H-NS binding sequences on the DNA can direct the assembly of distinct three-dimensional nucleoprotein structures.

Why does H-NS form more extended bridged filaments on PUUP compared with the other constructs? The main difference between PUUP and the other constructs is the largest spatial separation between the two P(*proV* NRE) regions due to two contiguous copies of UAS sequences. Assuming that nucleation of an H-NS bridge requires an interaction of the H-NS dimer bound at a high-affinity site with another DNA duplex (22, 28), the probability of independent engagement of distant secondary DNA sites would be higher for PUUP construct with the largest linear distance (941 bp) between the high-affinity H-NS binding sites. This notion is consistent with highest percentage of bridged *versus* non-bridged plasmids observed for PUUP (∼80%) compared with UPPU (∼56%) or UPUP (∼65%) (Tables 1 and 2). However, the high proportion of BL structures formed by PUUP construct (Table 1) would also be consistent with juxtaposition of the distant P elements forming a “hairpin” that extends with increasing H-NS concentrations.

Why does H-NS constrain higher levels of negative superhelicity on binding UPPU compared with the other constructs? The preference of DNA to writhe in a particular sense depends on its sequence. The sequence periodicity of the regulatory region of the *proV* NRE is ∼11 bp (>10.5 bp), whereas the sequence periodicity of the *tyrT* UAS is ∼10.2 bp (<10.5 bp), potentially encoding respectively right-handed (plectonemic) and left-handed (toroidal) coiling (43). On this assumption, the *proV* NRE and *tyrT* UAS sequences would, respectively, facilitate and impede DNA bridging by H-NS. This peculiarity of sequence organization would facilitate plectonemic coiling of duplexes and polymerization of H-NS bridges across the two contiguous “internal” P elements in UPPU. Indeed, the LBL structures favored on UPPU construct suggest a configuration in which the two adjacent *proV* NRE regions engaging all the four consecutive high-affinity nucleation sites are collinearly intertwined with the partner duplex. In contrast, the preference for toroidal coiling of the two contiguous U elements in PUUP would limit H-NS bridging across the internal U elements. This notion is fully consistent with the results of probing by restriction endonucleases showing the highest protection rate of restriction sites located inside the insert for UPPU construct as opposed to the PUUP construct, where higher protection is observed for restriction sites in the plasmid backbone (Fig. 8, *A–C*). Accordingly, the H-NS binding site 2 is more protected in UPPU than PUUP. Furthermore, H-NS-induced DNase I hypersensitivity between the two P elements in UPPU (Fig. 8*D*) is consistent with bending of the neighboring DNA induced by H-NS binding at thermodynamically unstable sequences of the high-affinity sites (22), which would reduce the DNA bending energy cost during plectoneme formation (34). We propose that cooperative bridging and bending of DNA by consecutive high-affinity sites of tandem *proV* NRE elements in UPPU facilitates accommodation of high negative superhelicity by stabilizing a narrow high pitch plectoneme (Fig. 6*B*).

It is assumed that the variable nucleoprotein structures stabilized by cooperative and antagonistic interactions between the NAPs mediate the communications between the environment and the genetic system of a cell (5, 17). For H-NS, diverse modes of DNA binding have been observed under the experimental variation of DNA supercoiling, ionic strength, temperature, and spatial confinement of nucleoprotein complexes (11, 12, 18, 21–23, 32, 44, 45). Furthermore, H-NS was proposed to form a right-handed superhelical protein scaffold that may dictate the DNA geometry in the complex (46). However, in this work we varied only the linear arrangement of otherwise identical *proV* NRE and *tyrT* UAS sequences. We infer that the DNA sequence organization, namely arrangement of regions with distinct sequence periodicity, dictates the properties of H-NS nucleoprotein complexes including their overall shape and the capacity to constrain supercoils and compact the DNA. On the example of H-NS, we thus demonstrate a DNA sequence-dependent potential for a NAP to increase the variability of chromosomal structures and, eventually, the topological differentiation of the bacterial nucleoid. To this end it is noteworthy that H-NS binding sites are non-randomly distributed in the genome (20, 28) and the degree of silencing by H-NS likely depends on the density of binding sites (47). Spatial organization of H-NS binding sites was implicated in graded inhibition of transcription (22) and determination of temporal order of gene expression (48), whereas binding of H-NS in intragenic regions has been implicated in genome-wide prevention of spurious transcription initiation events (49). To this end it is noteworthy that also in eukaryotes, a recent study implicated a distinct spatial organization of binding sites for the chromatin architectural protein CTCF in extrusion of chromatin loops (50). An intriguing question arising from our study is as to whether the various physiological effects of H-NS involve distinct nucleoprotein structures specified by differences in DNA sequence organization.

## Experimental procedures

### Constructs

The three ∼4-kb constructs UPUP (3992bp), UPPU (3997bp), and PUUP (3997bp) contained the bendable DNA sequences amplified from the UAS of the *tyrT* gene (denoted as U) and DNA with H-NS binding sites amplified from the NRE of *proV* gene (denoted as P) of *E. coli*. In these three constructs the individual U and P elements were cloned in different spatial arrangements. The positions of UAS sequences in U and of the two high-affinity H-NS binding sites in P are highlighted in bold italics: U(397bp), ctttgtttacggtaatcgaac***gattattctttaatcgccagcaaaaataactggttacctttaatccgttacggatgaaaattacgc***aaccagttcatttttctcaacgtaacactttacagcggcgcgtcatttgatatgatgcgccccgcttcccgataagggagcaggccagtaaaaagcattaccccgtggtggggttcccgagtaacaaaaaaacaacagcataaataaccccgctcttacacattccagccctgaaaaagggcatcaaattaaaccacacctatggtgtatgcatttatttgcatacattcaatcaattgttatctaaggaaatacttacatatggttcgtgcaaacaaacgcaacgaggctctacgaatcgagagtgcgt. P(264bp), gccacatttgccatcaggggttgcctcagattctcagtatgttagggtagaaaaaagtgactatttccattgggt***aatatatcga***catagacaaataaaggaatctttctattgcatggcaattaaattagaaattaaaaatctttataaaatatttggcgagcatccacagcgagcgttca***aatatatcga***acaaggactttcaaaagaacaaattctggaaaaaactgggctatcgcttggcgtaaaagacgccagtctggc).

In the first step the amplification of chromosomal DNA produced linear P fragments flanked by HindIII and XbaI restriction sites on the one side and BamHI site on the other as well as P fragments flanked BamHI site on the one and NheI and EcoRI sites on the other. The U fragments with flanking restriction sites were produced in similar way. The intermediate UP and PU constructs were generated by joining U and P sequences cleaved at the BamHI linker end, ligation, and cloning in pUC18 using the HindIII and EcoRI restriction sites. In a second cloning step UP and PU were amplified from the intermediate UP and PU constructs and joined via XbaI and NheI compatible sticky end ligation removing XbaI and NheI restriction sites and cloned in pUC18 using HindIII and EcoRI sites. In the final constructs all U and P sequences are arranged as direct repeats (Fig. 1).

### Analyses of topoisomer distributions

H-NS was purified as described previously (23). The PUUP, UPPU, and UPUP plasmid DNA (250 ng) was incubated with increasing H-NS concentrations in a buffer containing 12.5 mm Tris-HCl, pH 8.0, 50 mm NaCl, 2.5 mm MgCl_2_, 0.5 mm EDTA, and 100 μg/ml gelatin in a 20-μl volume for 30 min at 37 °C. Afterward, topoisomerase I of *E. coli* (a kind gift of M. Glinkowska) was added for another 30 min. The reaction was stopped by the addition of EDTA and phenol extraction. Purified plasmids were subjected to high resolution gel electrophoresis for analyses of topoisomer distributions of plasmids using 1× Tris borate-EDTA 1% agarose gels in the presence of different chloroquine concentrations as described previously (51).

### Sample preparation

A control sample without H-NS was first prepared for every DNA construct. The constructs were nicked using the Nt.BspQI nuclease (New England BioLabs) and purified from 1% agarose gel. DNA was diluted in the P1 buffer (1 mm Tris-HCl, 4 mm MgCl_2_, 0.003% Tween 20, 2.5% glycerol, pH 7.9) to a final concentration of 0.5 ng/μl, then a 20-μl aliquot of the mix was deposited on a freshly cleaved mica for 7 min. The mica was rinsed with 1 ml of ultrapure (18.2 megaohmns·cm resistivity) water and dried under a gentle flow of compressed filtered air. Several samples at different H-NS:DNA ratios were prepared for each DNA construct. H-NS was first diluted in the P1 buffer to desired concentration. Then the DNA was added to a final concentration of 0.5 ng/μl, and a 20-μl aliquot of the mix was left for incubation at 37 °C for 5 min. Afterward the mix was deposited on freshly cleaved mica for 7 min. The sample was then rinsed with 1 ml of ultrapure water and dried with a gentle flow of compressed filtered air.

### DNA deposition on APTES-modified mica surface

For the APTES deposition the mica surface was functionalized with APTES in a separate step before DNA deposition. Pure APTES (≥98% purity) was purchased from Sigma and diluted in ultrapure water to a final concentration of 0.1 volume %. A 15-μl droplet of diluted APTES solution was deposited on freshly cleaved mica for 1 min and then rinsed with 1 ml of ultrapure water and finally dried using a gentle flow of compressed nitrogen.

### AFM in air

AFM images were collected using a MultiMode SPM with a Nanoscope III controller (Veeco Instruments, Santa Barbara, CA) operated in tapping-mode in air. The AFM cantilevers used in air had a spring constant of 5 newtons/m (Bruker cantilevers, TAP150A) with resonance frequencies ranging between 120 and 160 kHz. All recorded AFM images consist of 512 × 512 pixels with scan frequency ≤1 Hz. Each H-NS-DNA binding experiment was performed at least in duplicate. AFM images were obtained at several separate locations across the mica surface to ensure a high degree of reproducibility and were used for statistical analysis of H-NS-DNA complexes. Only H-NS-DNA complexes that were completely visible in AFM image were considered for statistical analysis. The images were simply flattened using the Gwyddion software (Version 2.25) without further image processing (52).

### Ultra high vacuum AFM

Images were collected using a home built AFM operating at room temperature and in ultra high vacuum, as described elsewhere (53). Ultrasharp tips (NT-MDT) with nominal radii below 3 nm were driven at resonant oscillation frequencies (∼190–255 kHz) at 10-nm constant amplitude. All images were acquired at scan frequencies between 0.5 and 1 Hz. Images were collected using non-contact frequency modulation AFM operating mode, where a small negative frequency shift, typically of −2 Hz, was used as a feedback setpoint. This means that at all times during imaging no compressive force was applied to the sample, and only small attractive forces of ∼30 piconewtons were exerted by the AFM tip on the sample; this imaging procedure is identical to topographic imaging described previously (53). All images were flattened, and their height range was adjusted using Gwyddion software (Version 2.25) (52) without any further image processing.

### Analysis software

DNA molecules (typically 70–80 individual molecules) were analyzed using “DNA Trace,” Java-based analysis software that has been described elsewhere (54).

### Persistence length determination

We measured the persistence length *l_p_* of control and H-NS-bound DNA molecules by using the bond correlation function for polymers in two-dimensions,

$$<\;\cos\theta \left(s\right)\;>\;=e^{\left(-\text{s}/2\cdot \text{lp}\right)}$$

where θ is the angle between the tangent vectors to the chain at two points separated by the distance *s*, and *l_p_*, the persistence length (55).

### Probing with restriction enzymes

The cleavage reactions of the H-NS nucleoprotein complexes were carried out for 5 min with BamHI-EcoRI and BamHI-HindIII and for 10 min with ApaLI at 37 °C in the same standard New England BioLabs CutSmart buffer. H-NS was added in increasing concentrations to DNA constructs and incubated for 6 min at 37 °C before the addition of restriction enzymes. The reactions were stopped by the addition of 50 mm EDTA and 0.2% SDS (final concentration). The samples containing reaction products were loaded on agarose gels, and the intensities of individual DNA bands were analyzed using Gel analysis option on ImageJ software.

### DNase I footprinting

DNase I footprinting was performed in duplicates by using supercoiled plasmids at a final concentration of 20 nm and various H-NS concentrations (0–150 nm). The reaction buffer consisted of 40 mm HEPES, pH 8, 8 mm MgCl_2_, 60 mm KCl, 5 mm DTT, 0.05% Nonidet P-40, and 0.1 mg/ml BSA. The mixture was incubated at 22 °C for 20 min and 0.4 units of DNase I (Roche Applied Science) was added. Digestion was stopped with 1 volume of 50 mm EDTA after 30 s. After phenol/chloroform extraction, primer extension was performed using ^32^P-labeled primer P2 (5′-gccagactggcgtcttttacg-3′, annealing 72 bp downstream of H-NS binding site 2 in the P element) and Phusion DNA polymerase (New England BioLabs) following the manufacturer's instructions, with 20 cycles. The product was then loaded onto a sequencing gel. Sequencing reactions were performed as previously described (56).

DNA band intensity was quantified by using gel analysis feature on ImageJ software (25). For each analyzed band, the signal intensity of the area was measured and normalized to the total signal intensity in the entire track.

## Author contributions

A. J., S. R., P. S., L. S., and W. N. performed the experiments and analyzed the data. A. J., G. D., and G. M. designed the study, analyzed the data, and wrote the manuscript.

## Footnotes

5 The abbreviations used are:

NAP: nucleoid-associated protein
H-NS: histone-like nucleoid-structuring protein
NRE: negative regulatory element
UAS: upstream activating sequence
AFM: atomic force microscopy
APTES: 3-aminopropyl)triethoxysilane
BL: bridge-loop
LBL: loop-bridge-loop
*l_p_*: persistence length
*L_bf_*: bridged filament length.
